# Food intake restriction in patient with autism spectrum disorder and Moebius syndrome, a strong association. A case report

**DOI:** 10.1192/j.eurpsy.2024.942

**Published:** 2024-08-27

**Authors:** P. Del Sol Calderon, A. Izquierdo de la Puente, R. Fernández, M. García Moreno, A. Erdocia

**Affiliations:** ^1^Psychiatry, Hospital Puerta de Hierro; ^2^Psychiatry, Hospital Universitario Infanta Cristina, Madrid, Spain

## Abstract

**Introduction:**

A 7-year-old male diagnosed with autism spectrum syndrome and moebius syndrome was admitted to the psychiatric inpatient unit for a 3-week history of food restriction

**Objectives:**

To show the importance of exploring symptoms of autism in patients diagnosed with moebius syndrome in order to optimize the intervention of the difficulties that may arise.

**Methods:**

Case report and literature review

**Results:**

This is a patient with a history of Moebius Syndrome, who required trauma surgery for a clubfoot in April 2019. In early childhood he needs early psychomotor care. He has an IQ of 91. A diagnosis of autism was made in 2018 highlighting high difficulty for social interaction and communication, with repetitive patterns of behavior and marked restricted interests. The patient came to the emergency room after 3 weeks of food restriction. His parents explain that about a month ago the patient witnessed one of his classmates having an episode of vomiting. Since then he has been afraid that he might vomit. They explain that he constantly asks about food expirations, needing to ask before each meal if it will sit well in his stomach. He has noticeably decreased the amount of food he eats and is becoming more selective with food. In the last week he has lost 2 kilograms. During the hospitalization we worked with the patient on his fears about intakes, achieving a weight recovery and normalizing his eating habits.

**Conclusions:**

This case points out the association between Moebius syndrome and autism spectrum disorder. In addition, it reflects the importance of early diagnosis, since in this case it was essential to know the patient’s tendency to literalism and rigid thinking in order to receive effective treatment to achieve renutrition. Moebius syndrome is a rare congenital disorder with a prevalence of less than 0.05%, characterized by congenital facial paralysis associated with absence of abduction of the eyes due to alterations of the VI and VII cranial nerves. It presents multiple craniocephalic, musculoskeletal, neurological or ophthalmological manifestations. Different studies have found an association between autism spectrum disorder and Moebius syndrome, with comorbidity between 25-40%, varying according to the studies.

**Disclosure of Interest:**

None Declared

